# Iron is not everything: unexpected complex metabolic responses between iron-cycling microorganisms

**DOI:** 10.1038/s41396-020-0718-z

**Published:** 2020-07-20

**Authors:** Rebecca E. Cooper, Carl-Eric Wegner, Stefan Kügler, Remington X. Poulin, Nico Ueberschaar, Jens D. Wurlitzer, Daniel Stettin, Thomas Wichard, Georg Pohnert, Kirsten Küsel

**Affiliations:** 1 Institute of Biodiversity, Aquatic Geomicrobiology, Friedrich Schiller University Jena, Dornburger Strasse 159, 07743 Jena, Germany; 2 Institute of Inorganic and Analytical Chemistry, Friedrich Schiller University Jena, Lessingstr. 8, 07743 Jena, Germany; 3 Mass Spectrometry Platform, Faculty of Chemistry and Earth Sciences, Friedrich Schiller University Jena, Humboldstr. 8, 07743 Jena, Germany; 4 German Centre for Integrative Biodiversity Research (iDiv) Halle–Jena–Leipzig, Deutscher Platz 5e, 04103 Leipzig, Germany

**Keywords:** Biogeochemistry, Microbial ecology

## Abstract

Coexistence of microaerophilic Fe(II)-oxidizers and anaerobic Fe(III)-reducers in environments with fluctuating redox conditions is a prime example of mutualism, in which both partners benefit from the sustained Fe-pool. Consequently, the Fe-cycling machineries (i.e., metal-reducing or –oxidizing pathways) should be most affected during co-cultivation. However, contrasting growth requirements impeded systematic elucidation of their interactions. To disentangle underlying interaction mechanisms, we established a suboxic co-culture system of *Sideroxydans* sp. CL21 and *Shewanella oneidensis*. We showed that addition of the partner’s cell-free supernatant enhanced both growth and Fe(II)-oxidizing or Fe(III)-reducing activity of each partner. Metabolites of the exometabolome of *Sideroxydans* sp. CL21 are generally upregulated if stimulated with the partner´s spent medium, while *S. oneidensis* exhibits a mixed metabolic response in accordance with a balanced response to the partner. Surprisingly, RNA-seq analysis revealed genes involved in Fe-cycling were not differentially expressed during co-cultivation. Instead, the most differentially upregulated genes included those encoding for biopolymer production, lipoprotein transport, putrescine biosynthesis, and amino acid degradation suggesting a regulated inter-species biofilm formation. Furthermore, the upregulation of hydrogenases in *Sideroxydans* sp. CL21 points to competition for H_2_ as electron donor. Our findings reveal that a complex metabolic and transcriptomic response, but not accelerated formation of Fe-end products, drive interactions of Fe-cycling microorganisms.

## Introduction

Chemical communication is often the driving force of mutualistic interactions within microbial communities [1, 2]. The excreted metabolites can positively affect the growth of co-existing organisms by providing key metabolites produced by one partner and needed by the other [3–8]. The reciprocal benefits resulting from the excretion of metabolites indicate that while each partner invests metabolic resources, they both gain something from the other mutualistic partner [9–11]. Such mechanisms can not only control growth but also spatial distribution within the community in cases of heterogeneous environments. Synchronization of lifestyles is particularly important for mutualism interaction based on the partner’s end products for energy production [7, 12, 13].

Fe-cycling microorganisms are often found coexisting in nature. This is explained by the needs of both the ferrous iron [Fe(II)] oxidizer and ferric iron [Fe(III)] reducer to have sufficient exposure to electron donors and acceptors. For lithoautotrophic Fe(II)-oxidizers, including *Gallionella* sp. and *Sideroxydans lithotrophicus* ES-1, Fe(II) serves as an electron donor under micro-oxic conditions, thereby providing ample reducing equivalents for the assimilation of carbon [14–17]. Microbial Fe(II) oxidation facilitates the rapid precipitation of Fe(III) oxy-hydroxide nanoparticles [18, 19], which are preferred by anaerobic heterotrophic Fe(III)-reducers, such as *Shewanella* spp. or *Geobacter* spp., due to their small mineral size [20–23]. For Fe-cycling, at least temporary access to oxygen, or to oxidized compounds, is necessary for the renewal of the Fe(III) pool [14, 16]. In wetlands or peatlands, this renewal is mediated by water table fluctuations, bioturbation, or the release of oxygen via plant roots [24] which allows interaction between Fe-cycling microorganisms regardless of their different demands for O_2_. Production of Fe(III)-chelating ligands like riboflavins by *Shewanella* spp. or the presence of natural organic matter with Fe-complexing or electron shuttling properties can overcome spatial restrictions and speed up microbial activities [25, 26].

Interestingly, a shared homology is observed between the genes encoding the Fe(III) reduction machinery located at the cell surface in *S. oneidensis* and the genes encoding the Fe(II) oxidation machinery of *Sideroxydans lithotrophicus* ES-1 [17, 27]. In *S. oneidensis*, the Mtr machinery, comprised of the *mtrABC* gene cluster, is a porin–cytochrome complex in the outer membrane enabling the electron transport through the outer membrane via various hemes to Fe(III) [28, 29]. The MtoAB complex of *Sideroxydans* is homologous to MtrA and MtrB and functions in the same manner, forming a porin–cytochrome complex. Thus, we hypothesized that in parallel to the exchange of genetic information, additional mutualistic interactions based on the exchange of each partner’s exometabolites may have been established between these Fe-cycling partner organisms.

To elucidate these interaction mechanisms, co-culture systems are needed to facilitate downstream analytics on both the transcriptome and metabolome levels. But, co-cultivation of Fe-cycling microorganisms is very challenging due to their different lifestyles for O_2_. We selected two model microorganisms, the microaerophilic, autotrophic Fe(II)-oxidizer *Sideroxydans* sp. CL21 isolated from Schlöppnerbrunnen fen [15] and closely related to *Sideroxydans lithotrophicus* ES-1 [17], and the facultative anaerobic Fe(III)-reducer *S. oneidensis* isolated from Lake Oneida, NY [30]. First, we optimized incubation conditions that allow both partners to thrive simultaneously. The co-cultivation design allowed us to explore the influence of exposure to the partner’s end product for energy production by analyzing changes in 16S rRNA gene copies, RNA-seq analysis, and metabolomics. We hypothesized that co-cultivation will lead to an accelerated iron wheel (Fe(II) ↔ Fe(III)), because the Fe-cycling machinery would be the most affected during co-cultivation. In addition, we tested the effect of the exometabolome of one partner organism on the activity of the other.

## Materials and methods

### Bacterial strains and cultivation conditions

*Sideroxydans* sp. CL21 and *S. oneidensis* were cultivated in liquid supernatant exchange, monoculture, and co-culture incubations [15, 30–32] using ATCC medium 2672 (modified Wolfe’s minimal media (MWMM)) amended with 10 mM MES buffer (pH 6.3) unless otherwise noted. *Sideroxydans* sp. CL21 stock cultures grown in semi-solid gradient tubes containing 0.15% agarose-stabilized MWMM (Biozyme LE Agarose; Biozyme Scientific GmbH, Hessisch Oldendorf, DE) and zero-valent Fe (Fe^(0)^) were used as inoculum (1 mL inoculum 100 mL^−1^ media) in these incubations. Zero-valent iron powder is often used as the source of Fe(II) to cultivate Fe(II)-oxidizing bacteria [33], and we observed enhanced biomass during cultivation of Sideroxydans sp. CL21 in semi-solid gradient tubes, in comparison to FeS or FeCl_2_. Fe^(0)^ also provided the best Fe source for the liquid co-culture incubations. *S. oneidensis* overnight cultures were grown aerobically in Luria-Bertani medium. 1 mL was transferred into 125 mL serum bottles containing 60 mL MWMM amended with 18 mM lactate and grown aerobically at room temperature. 2 mL of MWMM-grown *S. oneidensis* was harvested after ~14 h (late exponential growth), centrifuged (10,000 *g*, 5 min), washed twice, resuspended in 2 mL MWMM, and used as inoculum (final concentration: ~2 × 10^5^ cells mL^-1^) for the above mentioned incubations.

### Monoculture and co-culture incubations

**Fig. 1:**
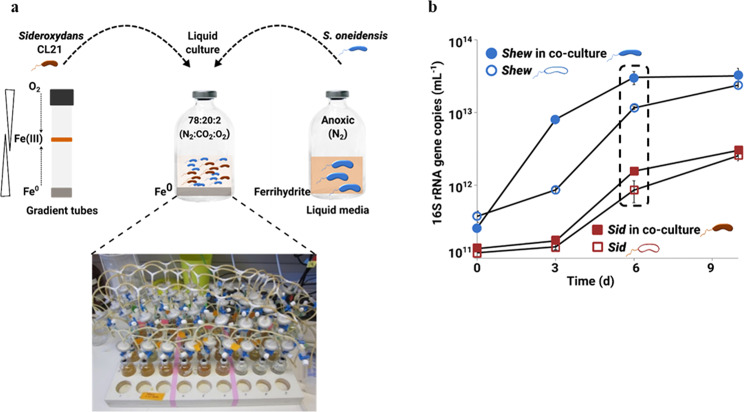
Co-culture incubation setup of *Sideroxydans* CL21 and *S. oneidensis* grown under microoxic conditions. **a** Pre-cultures were grown under standard optimized conditions either in gradient tubes or in liquid media prior to inoculation of co-culture incubations. **b** Both organisms can also grow in monoculture in this incubation set-up. 16S rRNA gene copy abundances of *Sideroxydans* CL21 or *S. oneidensis* showing growth in monoculture and in co-culture over 9 days of incubation. Data points represent mean abundances (*n* = 3) from either *Sideroxydans* CL21 monocultures (open squares) or *Sideroxydans* CL21 in co-culture (closed squares) and mean abundances (*n* = 3) from *S. oneidensis* monocultures (open circles) or *S. oneidensis* in co-culture (closed circles). In **b**, a box with dashed lines is used to indicate that samples from the 6 d time point were used for RNA-seq. Error bars represent standard deviation in triplicate incubations. In some cases, error bars are smaller than the symbols.

Monocultures and co-cultures were set up in triplicates in 125 mL serum bottles with an Fe^(0)^-containing bottom-layer plug (10 mL MWMM, 3% agarose (Biozyme LE Agarose), 1 g L^−1^ Fe^(0)^). Following solidification, 60 mL MWMM amended with 1 mM lactate was dispensed on top and continuously flushed with an N_2_:CO_2_:O_2_, 78:20:2, (flow rate: 300 mL min^−1^) gas mixture to maintain a micro-oxic environment (Fig. 1a). Cells were harvested at four time-points (6 h, 3 d, 6 d, 9 d), centrifuged) and stored at −80 °C. 6 d samples were selected for RNA-Seq.

### Cell-free supernatant exchange experiment

**Fig. 2:**
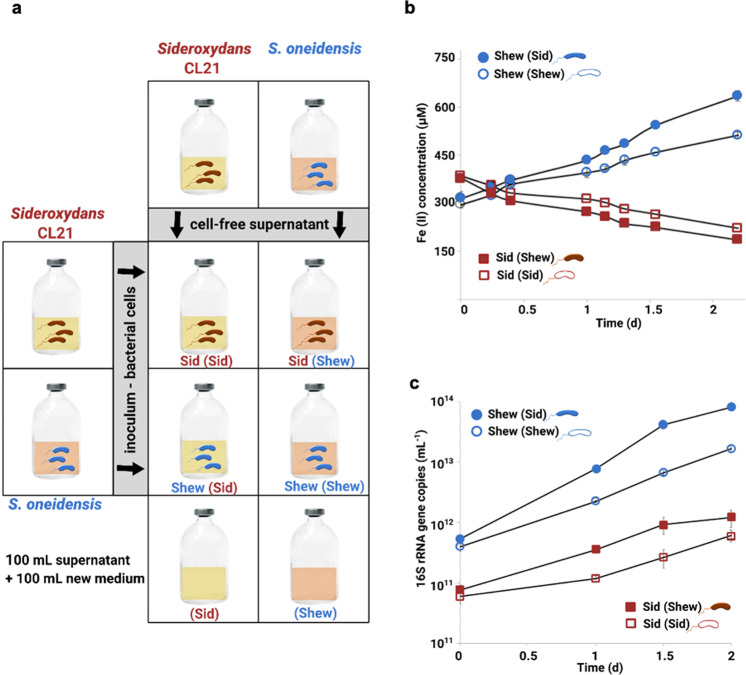
Cell-free supernatant exchange incubation setup, Fe(II) oxidation and Fe(III) reduction curves, and *Sideroxydans* CL21 or *S. oneidensis* 16S rRNA gene copies. **a** Briefly, monocultures of *Sideroxydans* CL21 (Sid) and *S. oneidensis* (Shew) were amended with cell-free supernatants obtained from either the same or partner strain. **b** Fe(II) concentrations were measured to follow Fe(II) oxidation in *Sideroxydans* CL21 incubations amended with either *Sideroxydans* CL21 [Sid (Sid)] or *S. oneidensis* [Sid (Shew)] cell-free supernatant or to monitor Fe(III) reduction in incubations with *S. oneidensis* amended with either *Sideroxydans* CL21 [Shew (Sid)] or *S. oneidensis* [Shew (Shew)] cell-free supernatant. **c** qPCR assays were used to determine mean abundances of 16S rRNA gene copies in triplicate samples from cell-free supernatant exchange incubations. Error bars represent standard deviation in triplicate incubations (*n* = 3). In some cases, error bars are smaller than the symbols.

Supernatant exchange experiments were set up in triplicates according to Mori et al. [34], with the following modifications: *Sideroxydans* sp. CL21 and *S. oneidensis* cultures were grown in 200 mL MWMM amended with 1 mM lactate and 1.2 mM FeSO_4_ or 10 mM hydrous Fe(III) oxyhydroxide, respectively, for 4 days with shaking at room temperature. Cultures were centrifuged, filtered (0.22 µm), cell-free supernatant was collected, and supernatant-exchange experiments were set up (1 mL inoculum 100 mL^−1^ media) (Fig. 2a). Fe(II) concentrations were measured using the phenanthroline method [35]. Cells were harvested at four time-points (0d, 1 d, 1.5 d, 2 d), centrifuged, and stored at −80 °C.

### Genomic DNA extraction

Cells were harvested via centrifugation (5000 *g*, 10 min, 4 °C), extracted using the DNeasy PowerSoil kit (Qiagen, Hilden, DE) according to manufacturer’s instructions, and frozen at −20 °C. *Sideroxydans* sp. CL21 genomic DNA was used for whole genome sequencing (PacBio sequencing) [36], and the assembled genome used as the reference genome for RNA-Seq analysis.

### Quantitative PCR

qPCR analysis using primer pairs Sid-120F/Sid-167R [18] or She12F/She220R was performed to determine total 16S rRNA gene copies of *Sideroxydans* sp. CL21 and *S. oneidensis* from supernatant exchange, monoculture, and co-culture incubations. 2–20 ng of genomic DNA was used as template for analyzes on a Mx3000P qPCR system (Agilent, Waldbronn, DE) with Maxima SYBR Green Master Mix (Thermo Scientific, Schwerte, DE). Standard curves produced using serial dilutions of representative plasmid mixtures (5 × 10^8^ to 5 × 10^2^ copies; *R*^2^ value = 0.999–1.000) were linear for both primer sets. The qPCR performed with efficiencies ranging from 80 to 90%.

### Sample prep for RNA-Seq analysis

Total RNA was extracted from three biological replicates from *Sideroxydans* sp. CL21 and *S. oneidensis* monoculture and co-culture incubations using the phenol-chloroform extraction method adapted from Wegner et al. [37]. Equimolarly pooled cDNA libraries were sequenced in paired-end mode (2 × 150 bp) on an Illumina NextSeq 500 platform by LGC Genomics (Berlin, DE).

### Transcriptome data processing and analysis

The quality of raw, demultiplexed RNA-Seq datasets was inspected using *FastQC* (v0.11.7) [38]. Quality trimming (settings: minlen=75, qtrim=rl, ktrim=rl, *k* = 25, mink=11, trimq=20, qtrim=rl) and adapter sequence removal was done with *bbduk* (v38.26) [39] using the included set of common sequence contaminants and adapters. rRNA-derived and non-coding RNA sequences were filtered out with *sortmerna* (v2.1) [40] and its pre-compiled *SILVA* databases [41] and *Rfam* [42]. The remaining, putatively mRNA-derived sequences were mapped onto the available *S. oneidensis* reference genome [43] and the newly sequenced and annotated *Sideroxydans*. sp. CL21 genome [36] using *bbmap* (v28.26) [39] (settings: slow, *k* = 11). Resulting bam files were sorted and indexed with *samtools* (v1.3.1) [44]. Genome annotations were used for generating simplified annotation format files for subsequent read counting (http://bioinf.wehi.edu.au/featureCounts/). Read counts (number of mapped reads per coding gene) were deduced from the generated bam files using the program *featureCounts* implemented in *subread* (v1.6.3) [45, 46]. The number of reads mapped to either *Sideroxydans* sp. CL21 and *S. oneidensis* in both monoculture and co-culture incubations can be found in Table S1. Differential gene expression analysis was carried out in the R framework for statistical analysis (v3.5.1) [47], using the package *edgeR* (v3.20.9) [48], including all dependencies.

## Metabolomics methods

### Sample preparation for metabolomic profiling

Sample preparation was conducted in triplicate as described previously [34], unless otherwise noted. Briefly, 50 mL of liquid cultures were centrifuged (4000 *g*) and extracted using Strata-X^®^ polymeric reverse phase cartridges (200 mg adsorbent; Phenomenex, Torrance, CA, USA). Sample preparation was performed in an anaerobic chamber to minimize Fe(II) oxidation and subsequent Fe(III) precipitation. (NOTE: in previous experiments, precipitation of Fe(III) during sample preparation resulted in completely dissimilar metabolite profiles—data not shown). Samples were measured in a randomized sample list immediately after preprocessing.

### GC/MS measurement

GC/MS metabolomics analysis was conducted on 3 and 6 d supernatant exchange samples using a high resolution Q-Exactive-GC electron impact (EI) orbitrap mass spectrometer (Thermo Scientific), with gas-chromatographic separations implemented on a Trace 1310 equipped with TriPlus RSH autosampler. A TG-5SILMS column (length = 30 m; inner diameter = 0.25 mm, 0.25 μm film; Thermo Scientific) was used. Column operation parameters and GC-Orbitrap settings used are described in the supplementary methods.

### XCMS metabolomic analysis

The XCMS data processing was carried out using –cdf files, which were converted from the Thermo RAW-files using the Xcalibur 3.0.63 (Thermo Scientific) onboard file converter. Processing was carried out using XCMS Server version 3.01.01. Pre-defined settings were used for GC-measurement “Single Quad (matched filter),” except, retention time correction was removed [49]. Parameters used are listed in Table S2.

### UHPLC/HRMS measurement and spectra acquisition

Ultra high performance liquid chromatography (UHPLC) coupled with high resolution MS (HRMS) of both for untargeted and targeted (γ-aminobutanoic acid (GABA) and zinc) metabolites was performed using an UltiMate HPG-3400 RS binary pump (Thermo Scientific, Bremen, Germany) and WPS-3000 autosampler (Thermo Scientific) equipped with a 25 µL injection syringe and 100 µL sample loop. Detailed parameters used are described in the supplementary methods.

### GC/MS data processing and annotation

Using an in-house R-script [50], detected masses were deconvoluted via peak-shape comparison to potential metabolites and reduced in number to only include potential metabolites containing ≥20 fragments. Potential metabolites present in blanks and media controls (<fivefold increase or decrease relative to bacterial samples) were removed (Table S3). The remaining potential metabolites were log-transformed and auto-scaled via Metaboanalyst 4.0 [51] to yield heatmaps with the 15 most dysregulated metabolites. Unknown metabolites were identified by comparing fragmentation patterns to known compounds in the NIST/EPA/NIH EI Mass Spectral Library NIST 17 (copyright United States Department of Commerce) searched with the NIST Mass Spectral Search Program v.2.3a nd Golm Metabolome Database library [52]. Metabolites with match scores >700 were tentatively identified and compounds with multiple match score hits >600 were labeled by compound class. During the analysis of supernatant exchange samples, an *S. oneidensis* control (Shew(Shew)) sample (6 d) was also removed from the analysis due to errors during spectral profiling.

### UHPLC/HRMS data processing and quantification

Quan Browser (XCalibur 3.0.63) (Thermo Scientific) was used for peak detection and integration for GABA and a detected zinc compound with the following settings: mass tolerance = 10 ppm, peak detection algorithm=ICIS; smoothing points=1; baseline window = 40; area noise factor=15; peak noise factor = 10; peak high = 5%, tailing factor = 2.5 and peak detection method = nearest RT. Each biological sample was measured in triplicate using a randomized sample list. The presence of GABA in the samples was confirmed by comparison to GABA standards. In order to identify metalloenzyme complexes, for example zinc complexes, MICP was applied to the UHPLC/HRMS data of the supernatant extracts and the zinc compounds were identified based on the natural isotopic pattern. The single isotopes of the metal-complexes were resolved due to a resolution of 280.000 at *m*/*z* = 200, e.g., for ^64^Zn-, ^66^Zn-, ^67^Zn-, and ^68^Zn-complexes for one substance. The identification is based on the mass differences between the single isotopic peaks of the substance (with an error <1 ppm) and the intensity of the mass peaks according to the abundance of the natural zinc isotopes.

## Results

### Merging lifestyles of *S. oneidensis* and *Sideroxydans* sp. CL21

*S. oneidensis* can be grown easily anaerobically in liquid media with Fe(III) as a terminal electron acceptor. However, the microaerophilic *Sideroxydans* sp. CL21 prefers growing in so-called “gradient” tubes filled with semi-solid media, an Fe(II) source in the bottom, and O_2_ supplied in the headspace. Opposing Fe and O_2_ gradients allow growth at a depth with optimal O_2_ concentrations of 20–40 µM [53]. Microbial Fe(II)-oxidation leads to the formation of a distinct orange ring of precipitated Fe(III) (oxy)hydroxides. However, the semi-solid media poses complications for the envisioned metabolomics profiling. To identify metabolites produced and secreted by each individual partner, we had to establish a suboxic liquid media setup that enables the growth of both *Sideroxydans* sp. CL21 and *S. oneidensis* in monocultures and co-cultures. In total we tested 48 combinations of media, carbon sources, buffer type, and Fe sources, bottle sizes, and headspace conditions to land on the best setup for our incubations (Fig. 1a): MWMM supplemented with MES buffer, lactate, and Fe^(0)^ provided in a semi-solid media plug at the bottom, and a headspace of N_2_:CO_2_:O_2_ (78:20:2) that is constantly flushed through the bottles. Under these conditions, Fe^(0)^ reacts slowly with water forming Fe(II) and H_2_ (anaerobic corrosion) and the released Fe(II) is slowly abiotically oxidized to Fe(III). Consequently, both Fe(II) and Fe(III) are continuously provided, and microbial activity should speed up the production of each Fe species. Unlike other Fe minerals, Fe^(0)^ is not ubiquitous in environmental systems and Fe^(0)^ is most often applied to groundwater and soil environments for in situ remediation of metalloid-contaminated sites. However, amendment with Fe^(0)^ results in enrichment of Fe(III)-reducing and Fe(II)-oxidizing microorganisms [54–58].

### Exometabolome stimulates growth and activity of each partner

First, we performed supernatant exchange experiments (Fig. 2a). Amendments with the partner’s cell-free supernatant led to enhanced rates of the production and consumption of Fe(II). Rates of Fe(II) oxidation by *Sideroxydans* sp. CL21 over 2 days increased from 4.3 to 5.5 μM h^−1^ (*t*-test, *p* = 0.002) when amended with *S. oneidensis* cell-free supernatant and Fe(III) reduction rates of *S. oneidensis* increased from 4.9 to 7.3 μM h^-1^ (*t*-test, *p* = 0.007) following amendment with *Sideroxydans* sp. CL21 cell-free supernatant (Fig. 2b, Supplementary Table S4). 16S rRNA gene copy numbers increased one order of magnitude in *Sideroxydans* sp. CL21 [Sid(Shew)] and two orders of magnitude in *S. oneidensis* [Shew(Sid)] after 3 days of batch incubation compared with its control Sid(Sid) or Shew(Shew), respectively (Fig. 2c).

**Fig. 3:**
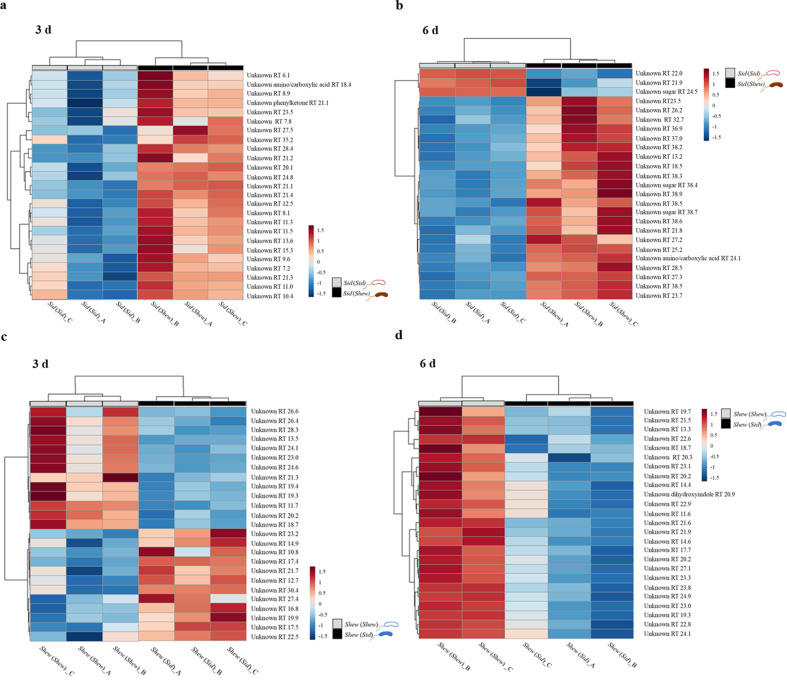
GC/MS based untargeted metabolome profiling of cell-free supernatant exchange experiments. Comparative analyses of the exometabolites produced by *Sideroxydans* sp. CL21 incubated with its’ own cell-free supernatant (50% *v/v*) (Sid(Sid)) versus *Sideroxydans* sp. CL21 incubated with the cell-free supernatant of *S. oneidensis* (50% *v/v*) (Sid(Shew)) after a 3 d (**a**) and 6 d (**b**) incubation period were conducted. Similarly, comparative analyses of the exometabolites produced by *S. oneidensis* incubated with its’ own cell-free supernatant (50% *v/v*) (Shew(Shew)) versus *S. oneidensis* incubated with the cell-free supernatant of *Sideroxydans* sp. CL21 (50% *v/v*) (Shew(Sid)) after a 3 d (**c**) and 6 d (**d**) incubation period were conducted. “_A, _B, or _C” corresponds to one replicate (*n* = 3).

These stimulatory effects show that diffusive metabolites are beneficial for this mutualistic relationship, such that metabolites from one species promotes growth and other metabolic processes, such as iron oxidation or reduction, of the other species. To identify potential chemical mediators, comparative (untargeted) metabolomic profiling was performed after 3 and 6 days. After three days of incubation with *S. oneidensis* supernatant, an increase in *Sideroxydans* sp. CL21 excretion was detected (Fig. 3a, Supplementary Table S3). A phenylketone was putatively identified by analyzing the accurate mass (309.0972 *m*/*z*) as a dehydrated fragment of a hydrated phenylketone with accurate mass (327.1077 *m*/*z*) via comparison with the NIST database (match scores of 631 and 673 respectively). The accurate mass of the dehydrated fragment fits within 1.4 ppm error according to exact mass. After 6 days of incubation with *S. oneidensis* cell-free supernatant, 88% of the top 25 dysregulated metabolites, meaning metabolites with concentrations affected by treatment, (out of 473 annotated peaks), were more abundant in the exometabolome of *Sideroxydans* sp. CL21 exposed to *S. oneidensis* supernatant (Fig. 3b, Supplementary Table S3). *S. oneidensis* exposed to the supernatant of *Sideroxydans* sp. CL21 also exhibited increased excretion with a total of 321 annotated compounds. A mixed metabolic response was observed, with only 52% of the top 25 most dysregulated compounds more abundant after 3 days. After 6 days, all 25 of the top dysregulated compounds were less abundant in *S. oneidensis* exposed to *Sideroxydans* sp. CL21 supernatant suggesting that these metabolites were not being produced at similar rates as in monocultures. For example, a dihydroxyindole was putatively identified (accurate mass 365.1655 *m*/*z*, which fits −0.8 ppm according to exact mass; NIST database R. match 674 and match score 658). An overall decrease in excretion at day 6 relative to day 3 was also exhibited, as evidenced by only 151 annotated compounds detected at day 6. Efforts to identify additional specific metabolites whose concentrations differed most were unsuccessful, which is in accordance with a high degree of secondary metabolite production that are not annotated in standard libraries (Fig. 3, Supplementary Table S3).

### RNA-Seq profiling of co-cultures show unexpected patterns of differentially expressed genes

*Sideroxydans* sp. CL21 and *S. oneidensis* were grown together over 9 days to cover potential mutually induced responses and effects of physical contact with the partner (Fig. 1a). Co-culturing was beneficial to growth, as the increase in 16S rRNA gene copies of both organisms was higher in co-cultures compared with monocultures (Fig. 1b) with the highest increase between 3 and 6 days. Thus, maximal 16S rRNA gene copies in co-cultures coupled with increased Fe-cycling rates in supernatant exchange experiments (Table S4) led to the selection of day 6 for RNA-Seq analyses.

**Fig. 4:**
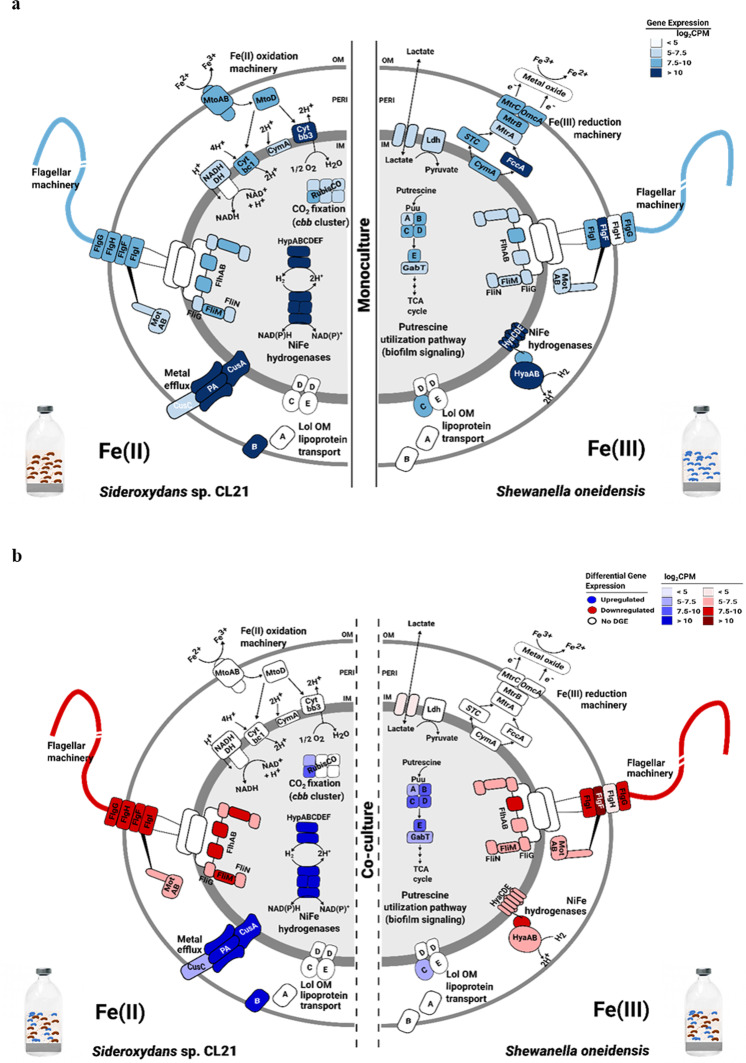
RNA-seq profiling reveals key metabolic pathways driving interactions in co-culture. Graphical representation of overall gene expression in *Sideroxydans* sp. CL21 and *S. oneidensis* monocultures (**a**) compared wiith the differential gene expression during co-cultivation **b** are shown. Differentially expressed genes were determined by analysis of RNA-seq data from triplicate co-culture (*Sideroxydans* CL21 + *S. oneidensis*) and monoculture batch incubations at the 6 d time point (see Fig. 1). log_2_CPM (CPM = counts per million) values represent a normalized measure for gene expression based on the mRNA-derived sequences that were mapped onto genes.

The overall transcriptome profiles of the monocultures demonstrated that genes involved in Fe(II) oxidation (i.e., the *Sideroxydans* sp. CL21 *mto* gene cluster) or Fe(III) reduction (i.e., the *S. oneidensis mtr* gene cluster) were highly expressed (ranging from 6.2–9.5 log_2_CPM), showing that not only *Sideroxydans* sp. CL21, but also *S. oneidensis* were utilizing Fe under suboxic conditions. Both monoculture gene expression profiles were dominated by genes encoding proteins linked to core metabolic functions, ribosomal proteins, RNA polymerase subunits, and ATP synthase subunits (Fig. 4a, Supplementary Tables S5, S6). Genes encoding a lactate permease and lactate dehydrogenase were highly expressed, which indicated *S. oneidensis* is using lactate as the electron donor (Supplementary Table S5). Genes involved in flagellar motility were also highly expressed in both organisms (Supplementary Tables S5, S6). In the case of *S. oneidensis*, we detected notably high gene expression values (7.5–9.0 log_2_CPM) for genes of the putrescine degradation pathway (Table 1, Supplementary Table S5). In the *Sideroxydans* sp. CL21 gene expression profiles, we identified multiple genes for cation efflux system proteins that featured particular high gene expression values (log_2_CPM > 10) (Supplementary Table S5). A cluster of NiFe-hydrogenases-related genes, including genes for hydrogenase subunits and proteins involved in hydrogenase assembly and maturation was uniformly and highly expressed with log_2_CPM values ranging between 7 and 10 (Table 2, Supplementary Table S6). Genes involved in the Calvin–Benson–Bessham pathway, which reduces 3-phosphoglycerate via RubisCO (ribulose-1,5-bisphosphate carboxylase/oxygenase), were highly expressed in *Sideroxydans* sp. CL21, such that genes encoding the proteins CbbO and CbbQ were upregulated (log_2_FC 1.02–1.31). The presence of both the small and large forms of RubisCO suggests a high tolerance to fluctuating O_2_ concentrations [17]. Also, genes encoding a lactate dehydrogenase and permease were highly expressed, thus implicating possible simultaneous use of inorganic and organic carbon during co-cultivation (Supplementary Table S6).

**Table 1:** Selected differential gene expression changes in *S. oneidensis* in co-culture versus monoculture.

Extracellular electron transfer pathways and accessory genes			
Gene ID	Gene	Function	Log_2_FC (fold change)
SO_0168	*gspF*	T2aSS secretion system inner membrane platform protein	1.18
SO_0396	*frdC*	Quinol-fumarate reductase menaquinol-oxidizing subunit	−2.19
SO_0397	*frdC*	Quinol-fumarate reductase menaquinol-oxidizing subunit	−1.02
SO_1778	*mtrC*	Extracellular iron oxide respiratory system surface decaheme cytochrome c component	−1.55
SO_4591	*cymA*	Membrane anchored tetraheme cytochrome c	−1.65
Polyamine Production/Degradation Genes			
SO_1274	*puuB*	Gamma-glutamylputrescine oxidoreductase	2.49
SO_1275	*puuC*	Gamma-glutamyl-gamma-aminobutyraldehyde dehydrogenase	2.32
SO_1276	*puuE*	GABA aminotransferase PLP-dependent	1.67
SO_3030	*pubA*	Putrescine monooxygenase	1.11
SO_4136	*speC*	Ornithine decarboxylase	−1.07
ABC-type Transport System Genes			
SO_0486	*nosD*	ABC-type copper transport system substrate-binding component	−1.05
SO_0565	*znuA*	ABC-type zinc uptake system substrate-binding component	1.17
SO_0566	*znuB*	ABC-type zinc uptake system permease component	1.20
SO_1044	*artI*	ABC-type arginine uptake system substrate-binding component	−1.05
SO_3714		ABC-type monosaccharide transport system substrate binding component	−1.11
SO_4318	*aggC*	Type I protein secretion system bifunctional ATPase and permease component	1.04
SO_4720	*tupB*	ABC-type tungstate uptake system permease component	1.55
Motility/Biofilm Formation Genes			
SO_0168	*gspF*	T2aSS secretion system inner membrane platform protein	1.18
SO_3350	*pilU*	Pilus retraction ATPase	−1.45
SO_3915	*cheX*	Chemotaxis signal transduction system CheY dephosphorylase	−1.49
SO_4053		Methyl-accepting chemotaxis protein	−1.16
Hydrogenase Genes/Energy Metabolism			
SO_2092	*hypC*	NiFe hydrogenase assembly chaperone	−1.92
SO_2093	*hypB*	Ni^2+^-binding GTPase involved in regulation of NiFe hydrogenase expression/maturation	−1.50
SO_2097	*hyaC*	Periplasmic [Ni-Fe] hydrogenase cytochrome b subunit	−1.04
SO_2096	*hyaD*	NiFe hydrogenase maturation protease	−1.28
SO_2099	*hyaA*	Periplasmic [Ni-Fe] hydrogenase small subunit	−1.42

Samples were collected from biological replicates at the 6 d time point. All positive changes (upregulated genes) and negative changes (downregulated genes) shown in Table 1 correspond to differential gene expression patterns in *S. oneidensis* in co-culture in comparison to the gene expression in the monocultures. *p* < 0.05 was considered significant.

**Table 2:** Selected differential gene expression changes in *Sideroxydans* CL21 in co-culture versus monoculture.

Transport/Secretion genes			
Gene ID	Gene	Function	log_2_FC (fold change)
SidCL21_0513	*exbD*	Biopolymer transport protein ExbD/TolR	3.74
SidCL21_0918	*lolB*	Outer membrane lipoprotein component of lipoprotein transport system	2.23
SidCL21_1317		Efflux transport system, outer membrane factor (OMF) lipoprotein	0.65
SidCL21_0494	*cusA*	Cobalt-zinc-cadmium resistance protein; cation efflux system protein	0.73
SidCL21_1055	*gspE*	Predicted secretion system protein	−2.04
SidCL21_1061	*gspF*		−2.03
Hydrogenases			
SidCL21_0646	*hypA*	[NiFe] hydrogenase nickel incorporation protein	1.63
SidCL21_0647	*hypB*	[NiFe] hydrogenase nickel incorporation-associated protein	1.84
SidCL21_0649	*hypC*	[NiFe] hydrogenase metallocenter assembly protein	1.42
SidCL21_0650	*hypD*	[NiFe] hydrogenase metallocenter assembly protein	1.41
SidCL21_0651	*hypE*	[NiFe] hydrogenase metallocenter assembly protein	0.91
SidCL21_0648	*hypF*	[NiFe] hydrogenase metallocenter assembly protein	1.56
SidCL21_0636	*hyaB*	Uptake [NiFe] hydrogenase, large subunit	3.10
SidCL21_0637	*hyaC*	Uptake [NiFe] hydrogenase, cytochrome b subunit	3.06
SidCL21_0635	*hyaA*	Uptake [NiFe] hydrogenase, small subunit	2.93
SidCL21_0649	*hyaD*	Uptake [NiFe] hydrogenase, maturation protease	2.67
SidCL21_1157	*hybA*	Hydrogenase-2 operon protein	3.05
SidCL21_1156	*hybB*	Ni/Fe-hydrogenase 2 b-type cytochrome subunit	2.99
SidCL21_1155	*hybC*	Ni/Fe-hydrogenase 2 large subunit	2.85
SidCL21_1158	*hybO*	Ni/Fe-hydrogenase 2 small subunit	2.79
Dehydrogenases			
SidCL21_0230	*fdsA*	NAD-dependent formate dehydrogenase alpha subunit	−1.79
SidCL21_0231	*fdsB*	NAD-dependent formate dehydrogenase beta subunit	−3.67
SidCL21_0232	*fdsG*	NAD-dependent formate dehydrogenase gamma subunit	−1.68
SidCL21_0739		NAD(P)H dehydrogenase	−0.82
SidCL21_2320	*ykgG*	Predicted l-lactate dehydrogenase, hypothetical protein subunit, Iron-sulfur cluster-binding subunit	1.62
SidCL21_2321	*ykgF*	Predicted l-lactate dehydrogenase, iron-sulfur cluster-binding subunit	1.36
SidCL21_2319	*ykgE*	Predicted l-lactate dehydrogenase, Fe-S oxidoreductase subunit	1.11
Flagellar/Motility/Biofilm Formation Genes			
SidCL21_3027	*fliE*	Flagellar hook-basal body complex protein	−0.81
SidCL21_0327	*cheY*	Chemotaxis regulator	−0.67
SidCL21_3038	*flgD*	Flagellar basal-body rod modification protein	−0.62
Extracellular Electron Transfer Pathways			
SidCL21_0020	*wrbA*	Multimeric flavodoxin	−0.86
SidCL21_0462		malate:quinone oxidoreductase	2.14
SidCL21_2568		quinolinate synthetase (EC 2.5.1.72)	1.80

Samples were collected from biological replicates at the 6 d time point. All positive changes (upregulated genes) and negative changes (downregulated genes) shown in Table 2 correspond to differential gene expression patterns in *Sideroxydans* sp. CL21 in co-culture in comparison to the gene expression in the monocultures. *p* < 0.05 was considered significant.

For co-culture incubations, 15.7 and 21.3% of the total genes were differentially expressed in *Sideroxydans* sp. CL21 and *S. oneidensis*, respectively. 927 genes were differentially expressed of which 428 were up- and 499 down-regulated in *S. oneidensis*, compared with 588 genes (249 up-, 338 down-regulated), which were differentially expressed in *Sideroxydans* sp. CL21 when grown in co-culture (Supplementary Fig. S1). Surprisingly, genes involved in Fe(II) oxidation or Fe(III) reduction in both partners were not upregulated in co-culture, but were still highly expressed, indicating growth in steady-state (Tables 1, 2; Supplementary Tables S5, S6). The most upregulated genes in *S. oneidensis* include genes encoding putrescine breakdown and an EPS biosynthetic gene cluster, as well as substrate-binding and permease components of zinc and tungstate ABC-type uptake systems. The most downregulated genes encode proteins linked to pilus retraction, type IV pili, and chemotaxis signal transduction (Table 1, Supplementary Table S5). In *Sideroxydans* sp. CL21, the most upregulated genes code for biopolymer and lipoprotein transport, NiFe hydrogenase incorporation and metallo-center assembly proteins, and hydrogenase-1 and -2 components. The first couples H_2_ oxidation in the periplasm to reduction of quinones in the inner membrane, whereas the second is involved in O_2_ reduction. Motility-related genes and genes associated with NAD-dependent formate dehydrogenase alpha, beta, gamma, and delta subunits and the sulfur carrier protein FdhD were among the most downregulated in *Sideroxydans* sp. CL21 (Table 2, Supplementary Table S6).

### Targeted candidate analyses

**Fig. 5:**
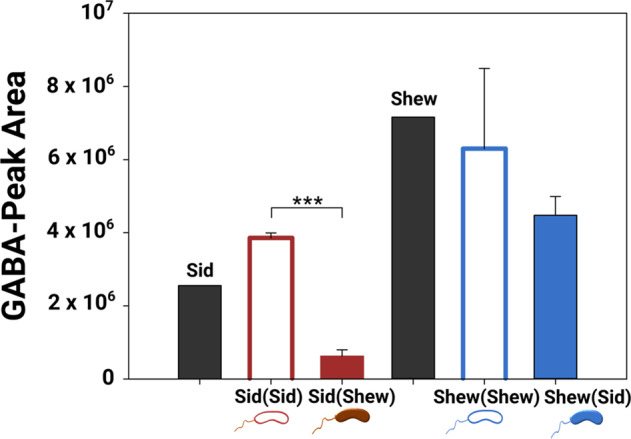
UHPLC/HRMS based targeted metabolomics profiling. Targeted metabolomics used to quantify the presence of γ-butanoic acid (GABA), a degradation product in the putrescine biosynthesis pathway, in *S. oneidensis* MR-1 and *Sideroxydans* CL21 supernatant exchange experiment. Sid and Shew correspond to *Sideroxydans* sp. CL21 and *S. oneidensis* monoculture control incubations, respectively. The labeling of Sid(Sid), Sid(Shew), Shew(Shew) and Shew(Sid) are described in the legend of Fig. 3. Area under the curve of GABA in the supernatant exchange experiment was measured on day 6. Error bars represent the standard deviation of three biological replicates (*n* = 3). Stars indicate significance: *p < 0.05, **p < 0.01 and ***p < 0.001.

Based on gene expression changes in *S. oneidensis*, which include gene clusters involved in putrescine and amino acid breakdown, an ABC-type tungsten- and an ABC-type zinc uptake system, we used targeted analyses to examine metabolites linked to these differentially expressed pathways. We detected GABA and evidence of ornithine and arginine [59, 60], all of which are metabolites comprising the putrescine biosynthesis and breakdown pathways. The upregulated pathways are thus responsible for the production of excreted intermediates, but putrescine itself and agmatine, another biosynthetic precursor, were not detected. We also detected GABA, ornithine, and arginine in the supernatant exchange experiment both on day 3 and day 6. We quantified GABA in supernatant exchange experiments on day 6 (Fig. 5). The amendment of the *Sideroxydans* sp. CL21 supernatant to *S. oneidensis* did not influence GABA amounts. In contrast, addition of the *S. oneidensis* supernatant to *Sideroxydans* sp. CL21 led to a significant decrease of GABA (*t*-test, *p* < 0.001, *n* = 3) (Fig. 5). No tungsten complexes were identified based on the evaluation of the isotope patterns of the molecular ions. Isotope pattern analysis indicated a metabolite containing complexed zinc. The identification of the metabolite containing complexed Zn was based on the mass differences between the single isotopic peaks of the substance (with an error <1 ppm) and the intensity of the mass peaks was determined according to the abundance of the natural Zn isotopes. Addition of *S. oneidensis* supernatant to *Sideroxydans* CL21 as well as the amendment of *Sideroxydans* CL21 supernatant to *S. oneidensis* led to reduced levels of the Zn compound (Fig. S2).

## Discussion

Systematic investigations of Fe-cycling microorganisms were hampered in the past due to their different requirements for oxygen. After methodical optimization, we could merge the growth requirements of a microaerophilic Fe(II)-oxidizer and an anaerobic Fe(III)-reducer to study interactions of organisms driving the complete iron wheel. The overall expression levels of genes involved in Fe(III) reduction in *S. oneidensis* and Fe(II) oxidation in *Sideroxydans* sp. CL21 were high under suboxic conditions. Although cell densities of standard monoculture incubations were not reached using this co-culture approach [61–64], we argue that such “candy shop” laboratory conditions do not mimic conditions in nature. Only co-culturing allows investigating the dynamics of interaction mechanisms of naturally co-occurring bacteria.

By combining transcriptomics and metabolomics, we disproved the hypothesis that the expression of genes involved in Fe(III) reduction and Fe(II) oxidation is the most affected during co-cultivation. Our data further suggest no acceleration of the iron wheel at all under these conditions. Excreted metabolites promoting growth and Fe-cycling were the greatest benefit of co-existence. Exometabolites from both *S. oneidensis* and *Sideroxydans* sp. CL21 elicit a positive effect on growth of their partners, suggesting a mutualistic interaction (Fig. 2). The physical presence of the partner seemed to be less important than the presence of the partner’s exometabolome.

Untargeted metabolomics revealed an overall upregulated metabolism of *Sideroxydans* sp. CL21 from day 3 to 6 when exposed to *S. oneidensis* exudates compared with *Sideroxydans* sp. CL21 alone (Fig. 3a, b). In contrast, *S. oneidensis* exposed to *Sideroxydans* sp. CL21 exudates showed a mixed response on day 3 and a predominately downregulated metabolism on day 6 (Fig. 3c, d). Here, the increased performance did not correlate with increased metabolite excretion. Alternatively, *S. oneidensis* might take up metabolites from the spent medium more efficiently if triggered with exudates from the partner. We found evidence for regulated phenylketone metabolism in *Sideroxydans* sp. CL21 exposed to *S. oneidensis* supernatant at day 3 and indole metabolism in *S. oneidensis* exposed to *Sideroxydans* sp. CL21 at day 6. Sugars, amino acids and carboxylic acids were differentially regulated at day 3, indicating a balanced response of primary metabolism by both partners.

The most differentially expressed genes of both partners grown in co-culture were involved in polyamine biosynthesis and degradation, biofilm formation, motility (i.e., flagella assembly and chemotaxis), utilization of inorganic and organic carbon substrates, and the formation of hydrogenases and dehydrogenases (Fig. 4b). These genes are known to be involved in physiological responses to environmental cues and nutrient availability, however, they are not uniquely related to Fe(III) reduction or Fe(II) oxidation.

The genomes of both *S. oneidensis* and *Sideroxydans* sp. CL21 encode NiFe hydrogenases, implicating a potential competition for H_2_. Periplasmic NiFe hydrogenases in *S. oneidensis* play a role in both H_2_ production and oxidation [65, 66], while the NiFe hydrogenases in *Sideroxydans* sp. CL21 enable only H_2_ oxidation [67]. In iron-rich peatlands, like the Schlöppnerbrunnen fen, H_2_ concentrations range from 4.5 to 6.0% in depths up to 40 cm [68, 69]. Here H_2_ appears to be produced by fermenters, with the rhizospheres providing a prime location, and is scavenged by anaerobic secondary fermenters, Fe(III) reducers, methanogens, and acetogens [68, 70–72]. As *Sideroxydans* sp. CL21 might be also able to utilize H_2_ in this fen as an alternative electron donor, variable oxygen concentrations released by plant roots in the rhizosphere and redox fluctuations due to variations in the water table might favor this microaerophile in the interspecies H_2_ competition. The differential gene expression patterns observed during co-cultivation confirmed interspecific H_2_ competition. Upregulation of genes encoding various NiFe hydrogenases in *Sideroxydans* sp. CL21 suggests the preferred utilization of H_2_, which serves here as an alternative electron donor to Fe(II). Simultaneous downregulation of genes encoding hydrogenases in *S. oneidensis* implicates synchronization between these Fe-cycling microorganisms. To compensate for the lack of H_2_ as the electron donor, *S. oneidensis* invests in accelerated lactate permease and dehydrogenase expression (Fig. 4b). These orchestrated responses in an environment offering multiple energy sources can even lead to a slowdown of the oxidative part of the iron wheel.

Differential gene expression patterns linked to biofilm formation suggest that *Sideroxydans* sp. CL21 and *S. oneidensis* communicate in co-culture using excreted metabolites to coordinate physiology. Biofilm formation was likely triggered by the upregulation of genes involved in the biosynthesis of putrescine and EPS, as well as the downregulation of genes involved in chemotaxis, motility, and type IV pili biosynthesis and activation in *S. oneidensis*. Detection of GABA, a metabolic product of putrescine, provides additional evidence that polyamines do play a role in microbial interactions [34]. In *Sideroxydans* sp. CL21, upregulation of genes involved in production and transport of biopolymers and lipoproteins and downregulation of genes involved in flagella machinery biosynthesis and motility are also relevant for biofilm formation during co-cultivation. Polyamine signaling in co-culture [73–75] might regulate biofilm formation activity of *Sideroxydans* sp. CL21. In conclusion, both partners invest in biofilm formation, thus initiating and supporting mutualistic interactions.

Polyamines, including norspermidine, spermidine, and putrescine are known to be required for cell growth and are important signaling molecules. Norspermidine enhances biofilm formation as an extracellular signaling molecule in *Vibrio cholerae* by interacting with a periplasmic sensor protein and a transmembrane protein [74, 76, 77]. Spermidine and putrescine transporters play a role in surface-associated growth of *Agrobacterium tumefaciens* and *Pseudomonas putida* [78, 79]. In *Yersinia pestis*, endogenous putrescine is required for the formation and development of biofilms. Amendment with putrescine rescues biofilm-deficient phenotypes in *Y. pestis*, implicating a role for both endogenous and exogenous putrescine in the activation of biofilm formation [80]. The latter finding is especially important for the current study, such that putrescine produced by *S. oneidensis* might play a dual role as an endogenous signal for biofilm development in *S. oneidensis* and an exogenous signal supporting the same process in *Sideroxydans* sp. CL21.

Other highly upregulated genes in *S. oneidensis* include those associated with ABC-type Zn transporters and ABC-type tungstate uptake systems (Table 1). Zn, an essential micronutrient, is also known to be toxic in excess as being a highly competitive divalent metal capable of displacing weakly bound transition metals in the active sites of metalloenzymes [81]. The observed upregulation of ABC-type Zn transporters suggests dynamic regulation of Zn homeostasis to preserve metabolic functioning. The upregulation of genes involved in ABC-type tungstate uptake systems hints that tungsten may function as a cofactor in place of molybdenum for formate dehydrogenases (FDHs) [82, 83]. Interestingly, FDHs can also use FeS clusters, flavins, or cytochromes as cofactors, all of which play a role in Fe(III) reduction and electron transfer [84]. While the links between tungsten-dependent enzymes and electron transfer in co-cultures are poorly understood, tungsten-containing FDHs are beneficial in certain syntrophic interactions under electroactive conditions [83].

*Sideroxydans* sp. CL21 and *S. oneidensis* share homologous Fe-cycling machinery: *mto* genes in *Sideroxydans* sp. CL21 and *mtr* genes in *S. oneidensis* [17, 27]. In both microorganisms, these gene clusters function as a porin–cytochrome complex forming an electron conduit through the outer membrane via various hemes. This cluster ultimately results in either reduction of Fe (III) outside the cell, coupled to the oxidation of organic carbon or H_2_ in case of *S. oneidensis* [28, 29], or the transfer of electrons from Fe(II) via MtoAB to a molybdoenzyme and subsequently to the respiratory chain (i.e., *cbb*_*3*_ or *bd* oxidase) via CymA [85] in *Sideroxydans* sp. CL21. The genes encoding MtoAB are located in a gene cluster also containing *mtoD*, a soluble periplasmic cytochrome encoding the protein MtoD, and *cymA* (homologous to *cymA* found in *S. oneidensis*), a quinol oxidoreductase encoding the protein CymA [17, 27]. The *mtoAB* cluster is also homologous to the *pioABC* gene cluster in *Rhodopseudomonas palustris*, which similarly forms a porin-cytochrome conduit [85].

Homologs to MtrAB and PioAB are widespread amongst Fe(III)-reducers and Fe(II)-oxidizers widely distributed across *Alpha-, Beta-, Gamma-*, and *Deltaproteobacteria* [85] pointing toward possible horizontal gene transfer events. It is believed that Fe(III) respiration is one of the oldest respiration processes on Earth [86] and aerobic microbial Fe(II) oxidation became possible billions of years ago when oxygen was released as a byproduct by photosynthetic organisms [85]. Various mechanisms to optimize mutual dependencies including communication might have developed over time when colonizing the same habitat despite seemingly different preferred ecological niches (i.e., microoxic vs. anoxic) [14, 87, 88]. Thus, the homologous Fe-cycling machinery, the ability of both bacteria to positively respond to the partner’s diffusive exometabolites concerning activity and growth, and the observation that gene expression is in flux (differentially expressed genes) when these microorganisms are active in co-culture, hints at potential co-evolution.

Our co-cultivation setup provides evidence that *S. oneidensis* is respiring Fe(III) under suboxic conditions without switching to its preferred terminal electron acceptor, O_2_. This flexibility allows adjustment to redox gradients and helps to explain the spatial co-existence of Fe-cycling bacteria in a variety of habitats [89–92]. Under fluctuating redox conditions in nature in which these microorganisms must continuously adapt, regulation of exometabolites can become key for Fe-cycling. Acceleration of the iron wheel via optimization of electron donor and electron acceptor availability may not be the most important factor driving their interaction, since Fe is the fourth most abundant element on Earth [93, 94]. Instead, both partners invest in shaping their environment via biosynthesis and detection of diffusive metabolites. Joint biofilm formation might be more important to ensure access to common goods, such as riboflavin, that function as electron shuttles [95–99]. High abundances of FeOB and FeRB have been reported from microbial mats, one of the most well-studied types of biofilms [100]. Simultaneous activity, which we mimicked in our setup, may not be as crucial in nature, where fluctuating redox conditions favor alternately one half of the iron wheel. Temporally decoupled redox processes are the rule and not the exception in nature.

## Conclusion

The results of this study have important implications for the understanding of microbial metal-cycling. Using an integrative transcriptomic and metabolomic approach, we revealed that this *Sideroxydans* sp. CL21 and *S. oneidensis* co-culture system can be used as a model system for future investigations focused on the interactions of Fe(II) oxidizers and Fe(III) reducers that likely occur in nature and the diffusive metabolites that play a role in these interactions. We provide quantitative evidence that *Sideroxydans* sp. CL21 and *S. oneidensis* benefit from growth in co-culture, and that diffusive metabolites play a more important role in biogeochemical processes than previously thought. Shaping the environment by a regulated inter-species biofilm formation appears to be the key mechanism underlying interactions of Fe-cycling microorganisms. Characterization of biofilms formed by *Sideroxydans* sp. CL21 and *S. oneidensis* during co-cultivation will provide further evidence about the regulation of these interactions, specifically in regards to producing, sharing, and utilizing common goods, such as diffusive chemical mediators and electron shuttles. Our data further demonstrated that competition for H_2_ between Fe-cycling partners provides another level of metabolic complexity when both partners are simultaneously active. Future incubation studies are needed to prove that *Sideroxydans* sp. CL21 does, in fact, prefer H_2_.

## Supplementary information

Supplemental Material

Supplementary Table S5

Supplementary Table S6

## Data Availability

The RNA-seq datasets used in this study have been deposited in ArrayExpress under the accession number E-MATB-9015 and the Metabolomics datasets have been deposited in MetaboLights under the accession number MTBLS1733.
