# Supplementary material for: Iron is not everything: unexpected complex metabolic responses between iron-cycling microorganisms
Source: ISME J. 2020 Jul 20;14(11):2675–90. doi: 10.1038/s41396-020-0718-z (PMC7784907; doi:10.1038/s41396-020-0718-z)
Supplement: Supplementary file 1 — Supplemental Material [file 41396_2020_718_MOESM1_ESM.docx]

**Supplementary Information for *Cooper et al*.**

**Iron is not everything: Unexpected complex metabolic responses between iron-cycling microorganisms**


Rebecca E. Cooper^1^, Carl-Eric Wegner^1^, Stefan Kügler^1,2^, Remington X. Poulin^2^, Nico Ueberschaar^3^, Jens D. Wurlitzer^1^, Daniel Stettin^2^, Thomas Wichard ^2^, Georg Pohnert ^2^, and Kirsten Küsel^1,4*^

^1^ Institute of Biodiversity, Aquatic Geomicrobiology, Friedrich Schiller University Jena, Dornburger Strasse 159, 07743, Jena, Germany

^2^ Institute of Inorganic and Analytical Chemistry, Friedrich Schiller University Jena, Lessingstr. 8, 07743, Jena, Germany

^3^ Mass Spectrometry Platform, Faculty of Chemistry and Earth Sciences, Friedrich Schiller University Jena, Humboldstr. 8, 07743, Jena, Germany

^4^German Centre for Integrative Biodiversity Research (iDiv) Halle-Jena-Leipzig, Deutscher Platz 5e, 04103, Leipzig, Germany

**Table of Contents**

Supplementary Materials and Methods……………..................………………………………….2

Supplementary Figures……………………………………………………………………………4

Figure S1………………………………………………………………………………......4

Figure S2…………………………………………………………………………………..5

Supplementary Tables…………………………………………………………………………… 6

Table S1…………………………………………………………………………………...6

Table S2…………………………………………………………………………………...7

Table S3…………………………………………………………………………………...8

Table S4…………………………………………………………………………………...9

Table S5………………………………………………………………………………….10

Table S6………………………………………………………………………………….10

**Supplementary Material and Methods**

**Sample preparation for RNA-Seq analysis**

Total RNA from triplicate *Sideroxydans* sp. CL21 and *S. oneidensis* monoculture and co-culture incubations was extracted using phenol-chloroform extraction method described by Wegner, et al (2019), with slight modifications. Briefly, cells were harvested at the 6 d time point via centrifuged (5000 *g*, 4 °C, 10 min). Cells pellets were washed in 15 mL falcon tubes with 5 mL 2x AE buffer (10 mM Tris-HCl, 0.5 M EDTA, pH 9) and 1.25 mL SDS (20% *v*/*v*) and vortexed (1 min) to facilitate detachment from Fe oxides. Following centrifugation (8000 *g*, 5 min, 4 °C), supernatants were transferred to new 15 mL Falcon tubes and extracted with phenol-chloroform-isoamyl alcohol (25:24:1) and chloroform-isoamyl alcohol (24:1). The subsequent supernatants were precipitated by adding sodium acetate (3 M, pH 5.2; final concentration 0.3 M), glycogen (final concentration 20 mg mL^-1^), and two volumes ethanol (100%) and incubated for 2 h at 4 °C. Precipitated samples were centrifuged (8000 *g,* 5 min, 4 °C) and pellets washed twice with ethanol (70%). Pellets were air dried and resuspended in RNase-free AE buffer. Total nucleic acids were quantified using the Qubit 3.0 Fluorometer (Thermo Scientific, Schwerte, DE) prior to digestion of DNA, and the quality of DNA checked via gel electrophoresis. RNA was enriched from resuspended nucleic acids by digesting genomic DNA with TurboDNase (Thermo Scientific, Schwerte, DE) according to manufacturer’s instructions. Total RNA was quantified using the Qubit 3.0 Fluorometer. rRNA was removed from total RNA extracts with Ribo-Zero rRNA Removal Kit (Illumina, San Diego, CA, USA) according to manufacturer’s instructions. Enriched mRNA was then quantified by chip-based gel electrophoresis using an Agilent Bioanalyzer 2100 (Agilent, Waldbronn, DE). Enriched mRNA libraries were prepared using minimum 5 ng of enriched mRNA and the NEBNext Ultra II Directional RNA Library Prep kit for Illumina (New England Biolabs, MA, USA) according to manufacturer’s instructions and purified with Ampure XP Beads (New England Biolabs, MA, USA). The resulting cDNA libraries were quantified by fluorometry and fragment size range assessed by chip-based gel electrophoresis using an Agilent Bioanalyzer 2100 and the Agilent DNA7500 kit (Agilent, Waldbronn, DE), equimolarly pooled, and sequenced in paired-end mode (2×150 bp) on an Illumina NextSeq 500 platform (LGC Genomics, Berlin, DE).

**Sample preparation for metabolomic profiling**

Sample preparation was conducted in triplicate as described previously [[39]](https://paperpile.com/c/Maz3Az/6uD5u), unless otherwise noted. Briefly, 50 mL of liquid cultures were centrifuged (4000 *g*) and extracted using Strata-X^®^ polymeric reverse phase cartridges (200 mg adsorbent, Phenomenex, Torrance, CA, USA). First, a conditioning step was performed by passing 2 mL UHPLC-grade methanol followed by an equilibration step of 2 mL UHPLC-grade water. Next, the complete sample broth was loaded on the SPE cartridges. 2 mL of UHPLC-grade water was used for elution for the LC/MS samples. For enrichment, each sample was evaporated to dryness in a nitrogen stream and subsequently re-dissolved with 100 μL of an aqueous 10 mM ammonium acetate solution. For GC/MS, the cartridges were dried by sucking the glove box atmosphere through the cartridge for 5 min. Cartridges were eluted with 2 mL acetonitrile/methanol 1:1 (*v*/*v*). The organic phase was evaporated under a N_2_ stream and re-dissolved in 50 µL tetrahydrofurane/methanol 1:1 (*v*/*v*). 25 µL was transferred into a 1.5 mL vial with insert and evaporated again. After drying in a desiccator overnight (20 mbar), 20 µL pyridine and *N*,*O*-bis(trimethylsilyl)trifluoroacetamide (BSTFA) were added, the samples were vortexed (30 s), and heated at 60 °C for 1 h.

**GC/MS measurement parameters**

Gas-chromatographic separations were implemented on a Trace 1310 equipped with TriPlus RSH autosampler and coupled with a Q-Exactive-GC electron impact (EI) orbitrap mass spectrometer (Thermo Scientific, Schwerte, DE). A TG-5SILMS column (Thermo Scientific, Schwerte, DE) with the following dimensions was used: length=30 m; inner diameter=0.25 mm, 0.25 μm film. The column was operated with helium carrier gas using a PTV injector with a column flow rate of 1 mL min^-1^ and injection for 1 min. The injector was operated initially at 60 °C, the temperature was then increased to 320 °C at a rate of 14.5 °C min^-1^, held for 2 min. For cleaning, the injector was heated to 350 °C for 5 min with a flow of 50 mL min^-1^. The split flow was set to 20 mL min^-1^. Following the sample injection, the syringe was washed five times with ethyl acetate and five times with *n*-hexane (5 µL each). The GC oven program began at 100 °C for 1 min and the temperature was increased to 320 °C at 5 °C min^-1^, and held for 3 min. The mass spectrometer began measuring after 10 min and monitored the mass range between 50 and 650 *m*/*z*. The MS transfer line and ion source temperature was set to 300 °C. Instrument settings: resolution=120 000 (FWHM); automated gain control (AGC target=1×10^6^; maximum inject time=“auto”; auxiliary temperatures=280 °C for transfer lines 1 and 2; EI source temperature=300 °C; EI was performed at 70 eV energy. N_2_ for supply of the GC Orbitrap C-Trap and HCD cell had a minimum purity of 99.999% (Linde AG, Munich, DE) and further dried using a moisture filter (the vendor specifies a gas quality of 6.0 after passage; Thermo Scientific).

**UHPLC/HRMS measurement and spectra acquisition**

Detailed parameters implemented for UHPLC/HRMS analysis using the UltiMate HPG-3400 RS binary pump (Thermo Scientific, Bremen, DE) and WPS-3000 autosampler (Thermo Scientific, Schwerte, DE) (25 µL injection syringe, 100 µL sample loop) are as follows: The column was kept at 25 °C within the column compartment TCC-3200. A Carbogen^®^ Zorbax SB-C8 chromatography column (150 × 4.6 mm; 1.7 µm; Agilent, Santa Clara, CA, USA) was used with the gradient specified in the Table S1. Eluent A contained water spiked with 2% acetonitrile and 1 mM ammonium acetate. Eluent B contained acetonitrile spiked with 10% water and 1 mM ammonium acetate. Mass spectra were recorded with QExactive plus orbitrap MS (Thermo Scientific, Schwerte, DE). Electrospray ionization was conducted in both negative (spray voltage=3300 V) and positive (spray voltage=3000 V) ionization modes. Electrospray ionization settings: Capillary temperature=360 °C; sheath gas flow=60; auxiliary gas flow=20; sweep gas flow=5; AGC-target=3.6 × 10^4^. Full scan analysis was conducted from *m*/*z* 100 to 1500 with a resolution of 280,000 at *m*/*z* 200 Measurements of γ-aminobutanoic acid (GABA) and zinc were conducted with the same parameters.


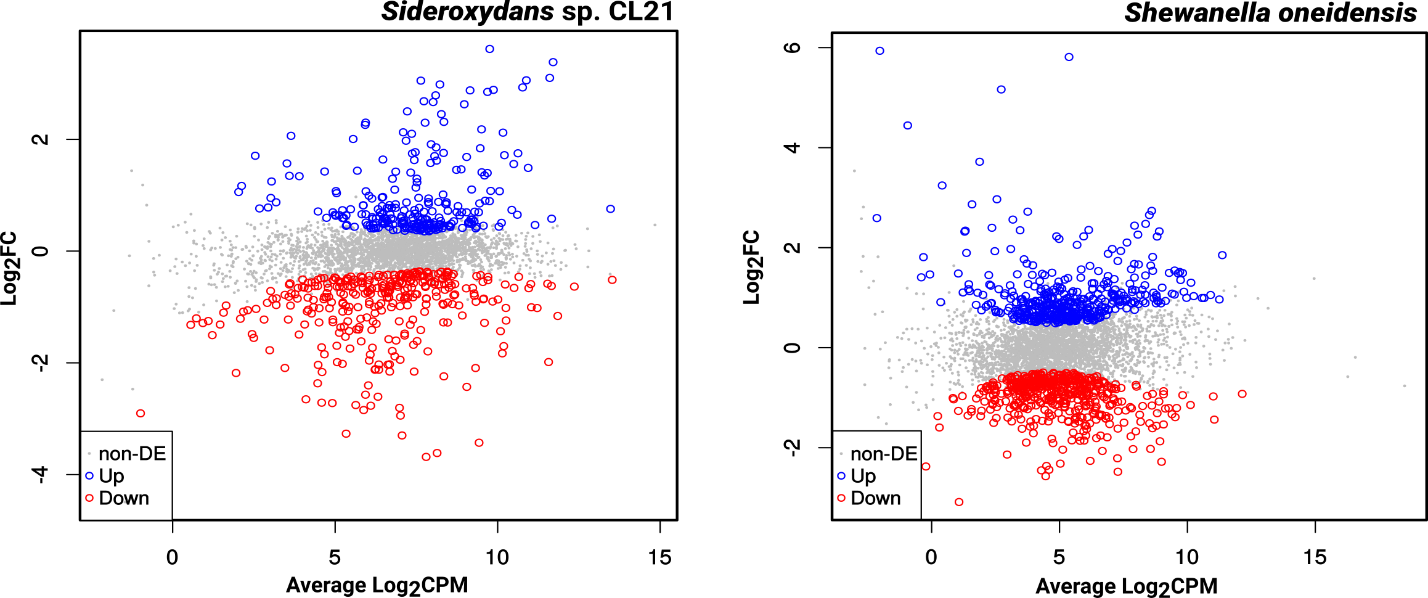


**Fig. S1.** The MA plots shown depict patterns of differential gene expression, specifically indicating highly expressed genes (non-DE (non-differentially expressed); grey) in comparison to upregulated genes (blue) and downregulated genes (red) in *Sideroxydans* sp. CL21 and *S. oneidensis* when grown in co-culture compared to monoculture incubations. Differentially expressed genes were determined by analysis of RNA-seq data from triplicate co-culture (*Sideroxydans* CL21 + *S. oneidensis*) and monoculture batch incubations at the 6 d time point (see Fig. 1). A log-fold change (log_2_FC) of 1 and p<0.05 significance cut-offs were used.

***
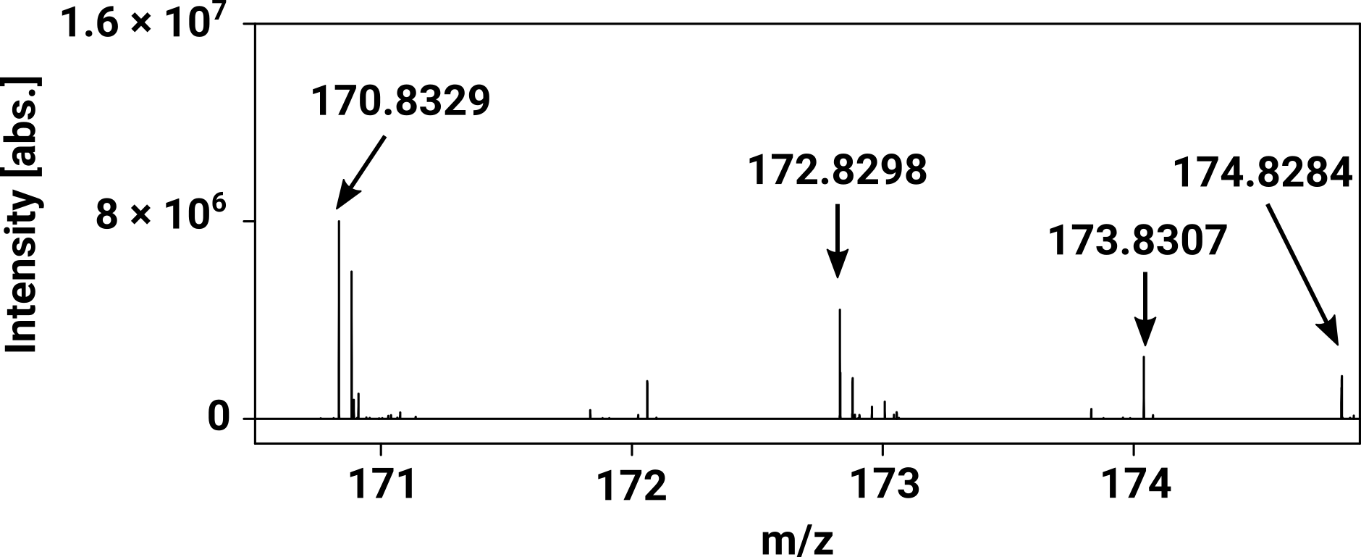
*a**

**
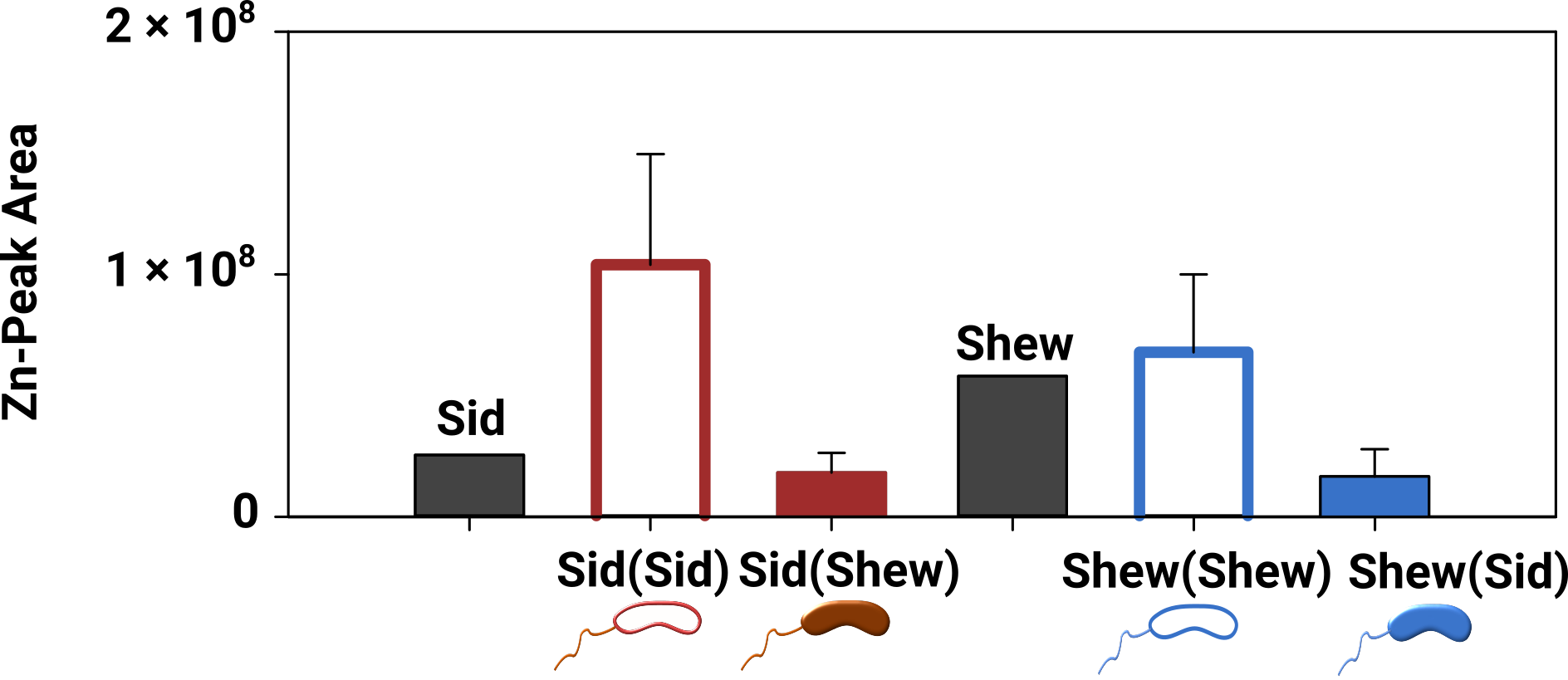
b**

**Fig. S2.** Zn compound in co-culture and supernatant exchange experiment with *Sideroxydans* CL-21and *S. oneidensis*. Mass spectra of the revealed Zn-compound depicts the appropriate Zn isotopic pattern **(a)**. Area under the curve for the supernatant exchange experiment of both *Sideroxydans* CL-21 and *S. oneidensis* after day 6 **(b)**. Note, the labeling of Sid(Sid), Sid(Shew), Shew(Shew) and Shew(Sid) are described in the legend of Fig. 3. Area under the curve of Zn (Zn-peak area) in supernatant exchange experiment was measured on day 6. Error bars represent the standard deviation of three biological replicates (n=3).

**Table S1.** RNA-Seq overview of monoculture and co-culture incubations. Values represent the averages of three replicates (n=3) for each incubation set-up (*Sideroxydans* CL21 monoculture; co-culture (*Sideroxydans* sp. CL21 + *S. oneidensis*); *S. oneidensis* monoculture). The number of mapped reads (Mapped *S. oneidensis*; Mapped *Sideroxydans* sp. CL21) is given for forward and reverse reads, respectively. Note, the lower percentage of reads mapped to the *Sideroxydans* sp. CL21 is due to either ambiguous mapping (i.e. a sequence mapped to two genes and the origin of the fragment is not clear) or sequences mapped to non-coding regions.

|  | ***Sideroxydans* sp. CL21** | **Co-culture** | ***S. oneidensis*** |
| --- | --- | --- | --- |
| **Raw** | 22187205 | 20934344 | 18286349 |
|  |  |  |  |
| **QC** | 19050215 | 17761629 | 15711982 |
| **[%]** | 85.7 | 84.8 | 85.9 |
| **Putative mRNA** | 18910660 | 17298817 | 14718289 |
| **[%]** | 99.3 | 97.4 | 94.2 |
| **Mapped *S. oneidensis*** | 47187 | 7670595 | 14613826 |
|  | 37616 | 7666895 | 14621646 |
| **[%]** | 0.3 | 44.2 | 99.3 |
|  | 0.2 | 44.2 | 99.3 |
| **Mapped *Sideroxydans* sp. CL21** | 7037602 | 3351482 | 14712 |
|  | 6884881 | 3298980 | 14862 |
| **[%]** | 37.5 | 19.3 | 0.1 |
|  | 36.7 | 19.0 | 0.1 |

**Table S2.** Parameters used for gradient for UHPLC / HRMS measurement.

| **Time [min]** | **Flow [mL min^-1^]** | **Concentration eluent B [%]** |
| --- | --- | --- |
| 0 | 0.4 | 0 |
| 0.2 | 0.4 | 0 |
| 4 | 0.4 | 50 |
| 4.2 | 0.4 | 100 |
| 5.2 | 0.4 | 100 |
| 6.2 | 0.4 | 0 |
| 6.5 | 0.4 | 0 |

**Table S3.** Metabolome profiling workflow parameters.

|  | **Supernatant Exchange Experiment (3d)** | | **Supernatant Exchange Experiment (6d)** | |
| --- | --- | --- | --- | --- |
|  | ***Shew*(*Shew*) vs.**  ***Shew*(*Sid*)** | ***Sid*(*Sid*)**  **vs. *Sid*(*Shew*)** | ***Shew*(*Shew*) vs. *Shew*(*Sid*)** | ***Sid*(*Sid*)**  **vs. *Sid*(*Shew*)** |
| **No. of mass/retention time pairs** | 26299 | 21514 | 23853 | 34803 |
| **No. of annotated compounds (AC)** | 875 | 659 | 743 | 667 |
| **No. of AC removed due to cartridge blank** | 130 | 21 | 170 | 124 |
| **No. of AC removed via supernatant (abiotic) control** | 424 | 524 | 422 | 70 |
| **Final no. of AC assumed to be metabolites of bacterial origin** | 321 | 114 | 151 | 473 |

**Table S4.** Rates of Fe(II) oxidation and Fe(III) reduction (μM h^-1^) in supernatant exchange experiments (See Fig. 2).

|  | **Fe(II) oxidation**  **(µm h^-1^)** | | **Fe(III) reduction**  **(µm h^-1^)** | |
| --- | --- | --- | --- | --- |
| ***Sid* (*Sid*)** | 4.3 | - | |  |
| ***Sid* (*Shew*)** | 5.5 | - | |  |
| ***Shew* (*Shew*)** | - | 4.9 | |  |
| ***Shew* (*Sid*)** | - | 7.3 | |  |

**Table S5.** Summary of RNA-Seq data of *S. oneidensis* grown in monoculture (Mono) or in co-culture (Co) with *Sideroxydans* sp. CL21 grown in liquid batch incubations. A, B, and C refer to data from three biological replicates. Total read counts are given for mono- and co-culture incubations. Overall gene expression is given in log_2_CPM. A, B, and C refer to data from three biological replicates. Gene expression values were also averaged (AVG) for both monocultures (AVG_Mono) and co-cultures (AVG_Co). Differential gene expression data of most downregulated and upregulated differentially expressed genes in *Sideroxydans* sp. CL21 grown co-culture with *S. oneidensis* compared to Sideroxydans sp. CL21 monocultures. Differential gene expression data is given in both log_2_FC (log_2_ fold change) and log_2_CPM (log_2_ counts per million) and represent averages of triplicate incubations (see corresponding .xls file).

**Table S6.** Summary of RNA-Seq data of *Sideroxydans* sp. CL21 grown in monoculture (Mono) or in co-culture (Co) with *S. oneidensis* grown in liquid batch incubations. A, B, and C refer to data from three biological replicates. Total read counts are given for mono- and co-culture incubations. Overall gene expression is given in log_2_CPM. A, B, and C refer to data from three biological replicates. Gene expression values were also averaged (AVG) for both monocultures (AVG_Mono) and co-cultures (AVG_Co). Differential gene expression data of most downregulated and upregulated differentially expressed genes in *Sideroxydans* sp. CL21 grown co-culture with *S. oneidensis* compared to *Sideroxydans* sp. CL21 monocultures. Differential gene expression data is given in both log_2_FC (log_2_ fold change) and log_2_CPM (log_2_ counts per million) and represent averages of triplicate incubations (see corresponding .xls file).
